# Use of data from various sources to evaluate and improve the prevention of mother‐to‐child transmission of HIV programme in Zimbabwe: a data integration exercise

**DOI:** 10.1002/jia2.25524

**Published:** 2020-06-30

**Authors:** Euphemia L Sibanda, Karen Webb, Carolyn A Fahey, Mi‐Suk Kang Dufour, Sandra I McCoy, Constancia Watadzaushe, Jeffrey Dirawo, Marsha Deda, Anesu Chimwaza, Isaac Taramusi, Angela Mushavi, Solomon Mukungunugwa, Nancy Padian, Frances M Cowan

**Affiliations:** ^1^ Centre for Sexual Health and HIV AIDS Research Harare Zimbabwe; ^2^ Liverpool School of Tropical Medicine Liverpool UK; ^3^ Organization for Public Health Interventions and Development (OPHID) Harare Zimbabwe; ^4^ London School of Hygiene and Tropical Medicine London UK; ^5^ University of California Berkeley Berkeley CA USA; ^6^ Ministry of Health and Child Care, Zimbabwe Harare Zimbabwe; ^7^ National AIDS Council, Zimbabwe Harare Zimbabwe

**Keywords:** PMTCT, PMTCT cascade, prevention cascade, data integration, data triangulation, data layering, HIV

## Abstract

**Introduction:**

Despite improvements in prevention of mother‐to‐child transmission (PMTCT) of HIV outcomes, there remain unacceptably high numbers of mother‐to‐child transmissions (MTCT) of HIV. Programmes and research collect multiple sources of PMTCT data, yet this data is rarely integrated in a systematic way. We conducted a data integration exercise to evaluate the Zimbabwe national PMTCT programme and derive lessons for strengthening implementation and documentation.

**Methods:**

We used data from four sources: research, Ministry of Health and Child Care (MOHCC) programme, Implementer – Organization for Public Health Interventions and Development, and modelling. Research data came from serial population representative cross‐sectional surveys that evaluated the national PMTCT programme in 2012, 2014 and 2017/2018. MOHCC and Organization for Public Health Interventions and Development collected data with similar indicators for the period 2018 to 2019. Modelling data from 2017/18 UNAIDS Spectrum was used. We systematically integrated data from the different sources to explore PMTCT programme performance at each step of the cascade. We also conducted spatial analysis to identify hotspots of MTCT.

**Results:**

We developed cascades for HIV‐positive and negative‐mothers, and HIV exposed and infected infants to 24 months post‐partum. Most data were available on HIV positive mothers. Few data were available 6‐8 weeks post‐delivery for HIV exposed/infected infants and none were available post‐delivery for HIV‐negative mothers. The different data sources largely concurred. Antenatal care (ANC) registration was high, although women often presented late. There was variable implementation of PMTCT services, MTCT hotspots were identified. Factors positively associated with MTCT included delayed ANC registration and mobility (use of more than one health facility) during pregnancy/breastfeeding. There was reduced MTCT among women whose partners accompanied them to ANC, and infants receiving antiretroviral prophylaxis. Notably, the largest contribution to MTCT was from postnatal women who had previously tested negative (12/25 in survey data, 17.6% estimated by Spectrum modelling). Data integration enabled formulation of interventions to improve programmes.

**Conclusions:**

Data integration was feasible and identified gaps in programme implementation/documentation leading to corrective interventions. Incident infections among mothers are the largest contributors to MTCT: there is need to strengthen the prevention cascade among HIV‐negative women.

## 1 INTRODUCTION

Although significant gains have been made in reducing mother to child transmission (MTCT) of HIV globally, there still remains an unacceptably high number of transmissions estimated at 170,000 and 160,000 new infections in 2017 and 2018 respectively; with all infected infants requiring antiretroviral therapy (ART) for life [1, 2] and facing an increased risk of significant morbidity and mortality that persists into adulthood [3]. To date very few countries have attained MTCT elimination status according to WHO validation criteria (≤50 new infections per 100,000 live births and a transmission rate of <5% in breastfeeding populations and <2% in non‐breastfeeding populations) [1, 4, 5]. Success in delivery of prevention of mother‐to‐child transmission (PMTCT) programmes is typically evaluated according to the PMTCT cascade – a series of sequential steps that need to be implemented to optimise care and prevention outcomes among HIV‐positive women, HIV‐negative women at risk of infection, and their babies [6]. Indicators to measure success have evolved over time in parallel with knowledge and WHO PMTCT guideline updates [6]. Although the importance of primary prevention among mothers has always been recognized (UNAIDS PMTCT prong 1) [7], to date cascade reporting has largely focused on MTCT outcomes (UNAIDS prongs 3 and 4). Given the growing proportion of MTCT occurring postnatally, with significant contribution from mothers who previously tested HIV negative [2], it is critical to pay attention to HIV prevention outcomes among pregnant or breastfeeding women in high prevalence settings. Both research studies and programme evaluations have documented losses to follow‐up at different steps along the cascades [8, 9, 10, 11]. The value of PMTCT cascade analysis for reporting PMTCT programme performance [12, 13], and for identifying gaps and appropriate interventions to strengthen quality of facility‐based PMTCT services [14, 15] is well established [6] and has informed use of other prevention, care and treatment cascades in the HIV field [11, 16, 17, 18, 19, 20].

The Global Plan to eliminate new HIV infections among children and keep their mothers alive (2010) [21] stimulated analyses of country PMTCT gaps and bottlenecks at each step of the cascade to strengthen programming. However, previous PMTCT cascade analyses have primarily utilised aggregate cross‐sectional data from routine programme reporting with known limitations and importantly, not reported HIV status of either HIV‐negative women in antenatal care (ANC) through delivery and postnatal period or exposed children at 18 to 24 months, or the proportion with HIV‐free survival at 24 months [6].

Together with implementing and research partners, Ministry of Health and Child Care (MOHCC) in Zimbabwe is tracking the progress towards elimination of MTCT (EMTCT) using a range of platforms. Although these data are shared, there has been no formal process to systematically integrate these data and maximise the learning they can provide. Public health triangulation/data integration is a process for reviewing, synthesizing and interpreting secondary data from multiple sources that bear on the same question to make public health decisions [22, 23].

In this study, we report on the process and results of a “data integration initiative” undertaken by MOHCC in partnership with implementing partners and researchers which aims to integrate data from different sources in order to give a fuller picture of performance of the national PMTCT programme. Results will be used to strengthen the impact of the PMTCT programme in Zimbabwe. Additionally, this process aims to identify data gaps required to inform programming and modelling across the region more broadly.

## 2 METHODS

The data integration working group comprising individuals from MOHCC, National AIDS Council, research and implementing partner organisations met biweekly from May to September 2019 to: (i) identify relevant data (ii) develop a system for integration (iii) develop cascades using integrated data (iv) identify data gaps (v) identify areas for programme improvement, and (vi) identify geographies/facilities for specific intervention.

### 2.1 Data sources

**Figure 1:**
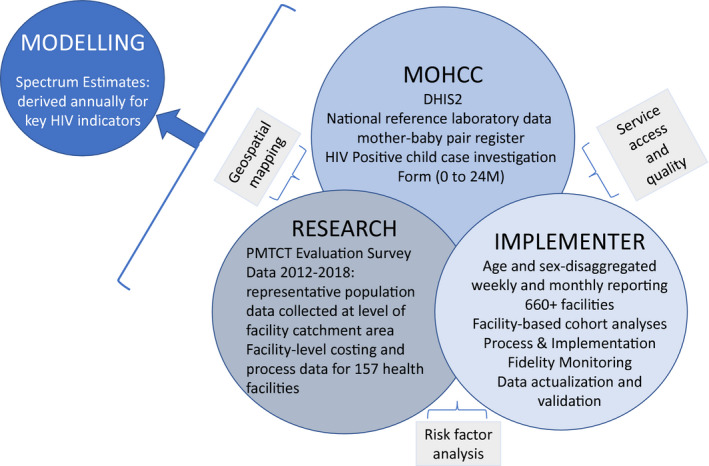
Data sources and domains.DHIS2, District Health Information System 2; MOHCC, Ministry of Health and Child Care; PMTCT, prevention of mother‐to‐child transmission.

We triangulate data from four sources: MOHCC, research, programme and modelling (Figure 1). Each source includes multiple types of data, providing individual, facility and population level evidence, with different strengths and weaknesses. For example, the research is population‐representative, used robust methods for data collection and cleaning, and, importantly, includes mother‐baby (MB) pairs who are lost to follow‐up from the health system. However, unlike programme data where health outcomes are verifiable on medical record, some survey outcomes are self‐reported. See Table 1 for detailed description of the data sources.

**Table 1:** Data sources and attributes for the integration exercise

	Research	Programme				Modelling
	PMTCT survey	DHIS2	PEPFAR DATIM	MB Pair register	Case investigation forms	Spectrum
Population	Representative, population level MB pairs 9‐18 and 19‐36 months	Attendees of health facilities	Attendees of health facilities	Attendees of health facilities	MB pairs where MTCT is recorded	
# Records (N)	2018: 7709 MB 9‐18 months; 1221 MB 19‐36 months 2012: 8800 2014: 10,404	448,475 women registered in ANC	177,706 women registered in ANC	Aggregate MB pair patient entries not documented	271 newly diagnosed infant‐HIV positive MB pairs	
Period covered	2017‐2018	2018‐2019	2018‐2019	2018‐2019	January 2018‐September 2019	
Data collection method	Population based survey in catchment areas of health facilities	Collation of data originally recorded on programme forms	Collation of data originally recorded on programme forms	Register completed longitudinally for each MB pair	Forms completed for each MTCT that is recorded	
Data type	Individual level	Aggregate at the facility level	Aggregate at the facility level	Individual level	Individual level	Population level
Geographical coverage	Catchment areas of 5 of 10 Zimbabwean provinces	National	669 health facilities in 24 districts	36 districts	669 health facilities in 6 Provinces	National estimate
Strengths	Robust data collection and cleaning Inclusion of MB pairs not currently in care (including if either M or B have died) Population‐representative estimates	National‐level data Objective data reporting using programme forms Monthly reporting for continuous performance monitoring Outcomes verifiable with source documents	Monthly reporting for continuous performance monitoring (support for rapid course correction) Outcomes verifiable with source documents	Longitudinal follow‐up of MB pairs	Detailed investigation of each MTCT	Population level impacts and outcomes
Weaknesses	Some outcomes are self‐reported Expensive Does not facilitate real‐time quality improvement	Aggregate cross sectional data Limited resources to validate/clean the data	Aggregate data	Paper registers with incomplete abstraction to electronic format	Incomplete data and low coverage (completed and entered form for each laboratory diagnosis)	Informed by programme data which may not be accurate/complete
Summary of data quality assessment	Good	Fair	Fair	Poor	Good	Fair

ANC, antenatal care; DHIS2, District Health Information System 2; MB, mother‐baby; MCTC, mother‐to‐child transmission; MOHCC, Ministry of Health and Child Care; PEPFAR/DATIM, President’s Emergency Plan for AIDS Relief/Data Accountability Transparency and Impact Measurement; PMTCT. prevention of mother‐to‐child transmission.

The data collection process for each is given below.

#### 2.1.1 Research

Between 2012 and 2018, researchers partnered with MOHCC to conduct an external evaluation of Zimbabwe’s PMTCT programme. Three representative, cross‐sectional, population‐based surveys were conducted in catchment areas surrounding the same 157 randomly selected health facilities in five of Zimbabwe’s ten provinces. Multi‐stage sampling was used to select facilities and MB pairs for inclusion. The study population consisted of infants born 9‐18 months before the survey and their biological mothers or caregivers aged ≥16 years old. Infants 9‐18 months old were selected to be able to detect HIV transmissions occurring during pregnancy, delivery and breastfeeding. Importantly, the survey aimed to include mothers or infants who had died since delivery, in which case verbal autopsy data were collected. All mother/caregiver participants completed an interviewer administered questionnaire and provided a dried blood spot sample for HIV testing. Details of survey methods have been published previously [24]. In 2017/2018, the survey was extended to include MB pairs where babies were 19‐36 months old specifically to explore retention of mothers and babies in the later post‐partum period. In depth data on PMTCT services offered at facilities were also collected.

#### 2.1.2 MOHCC

Through the National PMTCT Programme, MOHCC collects a wide range of programmatic data at 1560 health facilities across Zimbabwe into multiple paper registers that track engagement of mothers/infants at different cascade points including antenatal, delivery and postnatal service uptake and clinical outcomes. In 36 Districts, MOHCC has piloted the MB Pair register which tracks all MB (HIV positive and negative) from birth to 24 months. For each MTCT that is recorded, MOHCC recently introduced detailed case investigation and documentation of potential causes of transmission. This is recorded on paper then entered into a national database. At 624 high volume facilities, MOHCC enters data aggregated from Patient OI/ART Care Booklets into an Electronic Patient Monitoring System. Data entered onto monthly return forms are entered into the District Health Information System 2 (DHIS2) on a monthly basis with centralized data entry and retrieval at district level. MOHCC data are recorded by health workers who have a high workload and typically do not have time for routine quality assurance and data validation.

#### 2.1.3 Implementing partner

The local implementing partner, Organization for Public Health Interventions and Development (OPHID), has supported MOHCC with implementation of the National PMTCT Programme since 2001. Through President’s Emergency Plan for AIDS Relief (PEPFAR)/USAID funding, OPHID currently provides support at multiple health system levels to strengthen HIV Care and Treatment at over 660 health facilities in six Provinces through the Families and Communities for Elimination (FACE HIV) Programme. This support includes weekly, monthly and quarterly data collection and analysis of PEPFAR Data Accountability Transparency and Impact Measurement (DATIM) indicators, age‐ and sex‐disaggregated cross‐sectional service indicators, and targeted process and cohort‐based programme assessments. The data sources for OPHID’s programme mirror MOHCC data sources, but the frequency and granularity of targeted analysis is more intense. In addition, OPHID actively engages in data strengthening and strategic utilisation at health facilities through monthly facility‐level data consolidation and visualization activities, district level data triangulation meetings and annual data actualization.

#### 2.1.4 Modelling

United Nations Programme on HIV/AIDS (UNAIDS) supports national Programmes to make annual estimates of key HIV indicators. These estimates rely on national surveillance and survey data, national programme data as well as epidemic patterns derived from scientific studies. The Spectrum software is used to combine this information under specific assumptions to produce estimates of key indicators, including the number of people living with HIV by age and sex, new infections, AIDS deaths, AIDS orphans, the need for treatment and prevention, including PMTCT. In this study, we report Spectrum estimates on PMTCT outcomes.

### 2.2 Data handling and analysis

Drawing on previous work in data triangulation and evidence‐informed intervention design [22, 25], we followed four stages in the data triangulation: (1) Evidence attribute mapping; (2) Data quality assessment; (3) PMTCT Cascade Data Layering Analysis; and, (4) Data‐Driven Intervention Design. The cascade data layering analysis was conducted at geographic level rather than individual level because unique identifier data were not available for programme data.

#### 2.2.1 Evidence attribute mapping

We mapped the data to understand attributes of each source including defining the population, period covered, data collection methods, geographical coverage, and relative strength and weaknesses [23]. We then determined the availability of data for each step along an expanded PMTCT cascade that includes infant HIV status at 18‐24 months and HIV‐free survival [6].

#### 2.2.2 Data quality assessment

We assessed the relative strengths and weaknesses of each data source according to four major categories: conformance of data values to intended format and allowed values (e.g. for survey data we verified that numeric data, such as age, appeared as such, and we did additional checks if age of mother did not fall between 16 and 55 years. For programme data which were aggregate, examples of conformance checks included application of validation rules that numerators were smaller than denominators along the cascade and that historical trends in reporting were realistic with data verification for outliers). We also checked for completeness (extent of missingness), plausibility, for example verifying that dates of birth/delivery and early infant diagnosis made temporal sense, and relevance of data values. See Appendix for the framework that was used [26].

#### 2.2.3 PMTCT cascades data layering analysis

We constructed mother and infant cascades according to a comprehensive cascade framework [6], indicating engagement of (i) HIV‐positive women, (ii) HIV exposed infants and (iii) HIV‐negative women according to each of the four data sources. For the research we used data from the 2017/2018 survey. For MOHCC and OPHID data, proportions were calculated for 2018. We used the latest modelling estimates that were based on 2017/2018 data.

For each data source, we examined risk factors for poor programme performance such as poor health service uptake, ART initiation and MB retention using different methods according to available data. We only performed univariable analysis to determine risk factors for MTCT from survey data because there were too few MTCTs (25 in total) to conduct multivariable analysis. For the survey, we conducted a spatial analysis of MTCT hotspots using MTCT data. We layered this with MOHCC data on new positives so as to identify geographic target areas for enhanced prevention interventions. Risk factors for MTCT were further explored through analysis of detailed case investigation of MTCT that was supported by OPHID, and also through analysis of modelling outcomes.

Using patterns determined from geographic regions where data from all sources were available, we determined the feasibility and utility of extrapolating to regions/facilities with missing data. Although each data source was analysed individually, for the integration exercise we evaluated concordance in cascade indicator data across available sources, and identified information gaps and areas of poor performance across the PMTCT cascade.

### 2.3 Ethical considerations

The research (serial surveys) had ethical approval from Medical Research Council of Zimbabwe, reference numbers MRCZ/A/1655, MRCZ/A/ 1826, MRCZ/A/2162 for 2012, 2014 and 2017 surveys respectively. Approval was also obtained from the following ethics committees: University College London (2517/004), University of California Berkeley (2014‐02‐6038) and Liverpool School of Tropical Medicine (16‐063). Written informed consent was obtained from survey participants before study procedures were done. MOHCC and OPHID data were collected programmatically with verbal consent; with all but PMTCT case investigation (where names were necessary for follow‐up but was de‐identified at data entry and in generated reports) using deidentified data.

## 3 RESULTS

Table 1 shows a summary of the data that were available for triangulation. Data came from similar periods which made comparisons feasible, except for prior survey rounds which albeit provided important baseline and midline comparisons prior to HIV care and treatment guideline changes including Option B+ and Treat All. We recruited 8800; 10,404 and 7709 mother/caregiver infant dyads from the 2012, 2014 and 2018 surveys respectively. For women attending ANC, in DHIS2 and OPHID DATIM, 448,475 and 177,706, records were used respectively.

### 3.1 Engagement at different steps of the cascade

**Figure 2:**
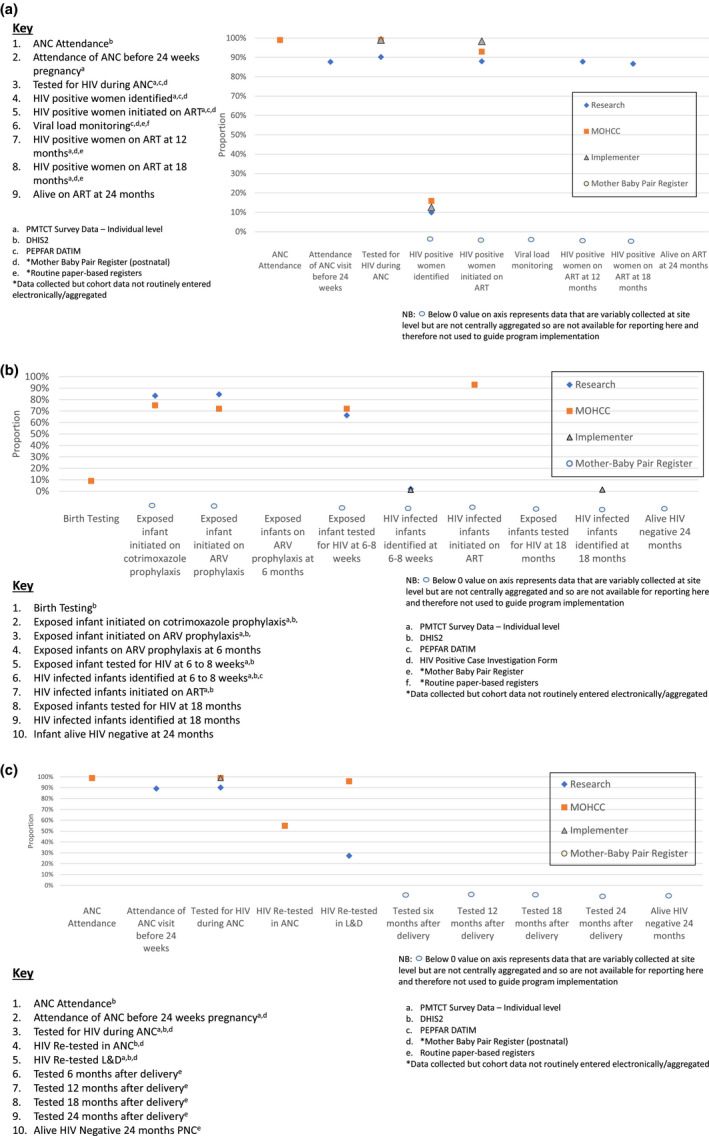
Cascade for (a) HIV‐positive women; (b) HIV‐exposed infants; (c) HIV‐negative women.ANC, antenatal care; ART, antiretroviral therapy; DHIS2, District Health Information System 2; MOHCC, Ministry of Health and Child Care; PEPFAR/DATIM, President’s Emergency Plan for AIDS Relief/Data Accountability Transparency and Impact Measurement; PMTCT, prevention of mother to child transmission.

We present separate cascades for HIV‐positive women (Figure 2a), HIV‐exposed infants (Figure 2b) and HIV negative women (Figure 2c) constructed using data from the four sources. Each cascade is constructed up to 24 months postnatal as median duration of breastfeeding, and consequent risk of MTCT in Zimbabwe (and elsewhere in the region) is 18 months. Overall, the different data sources showed similar trends, with data gaps later in all three cascades. The exception is for HIV testing in labour and delivery among eligible women. While available evidence on antenatal and perinatal cascade indicators is relatively complete, there are information gaps in all cascades during the postnatal period. For example, there are more data points from more sources for HIV infected women. There are few data on MTCT following early infant diagnosis (6‐8 weeks postnatal) in the infant cascade. While MOHCC has introduced a MB pair register to track individual outcomes of MB pairs from birth to 24 months, there are no reported data available for HIV‐negative mothers from any source in the post natal period resulting in poor understanding of coverage and outcomes of HIV re‐testing intended to identify maternal incident infection in the postnatal period. At present, cascade data on primary prevention for pregnant and lactating women testing HIV negative (i.e. referral and linkage rates of HIV‐negative women to HIV prevention services such as pre‐exposure prophylaxis (PrEP)) is completely lacking. Importantly, no data are routinely reported on the final outcomes for: HIV positive mothers alive on ART at 24 months (Figure 2a), HIV‐exposed infants at cessation of breastfeeding (Figure 2b) or among HIV negative women in the post‐partum period (Figure 2c). There are gaps in reporting viral load monitoring cascades among HIV‐positive pregnant and lactating mothers.

### 3.2 MTCT of HIV

**Figure 3:**
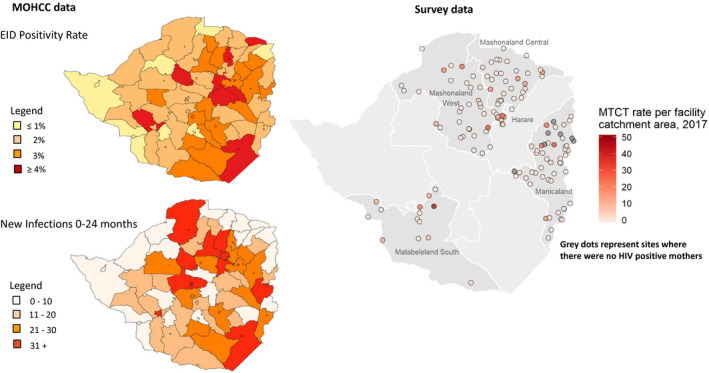
Spatial representation of MTCT across the country.MOHCC, Ministry of Health and Child Care; MTCT, mother‐to‐child transmission.

Survey data showed that MTCT by 9‐18 months post‐partum decreased from 9.5%, 5.1% and 3.4% in 2012, 2014 and 2018 respectively, while Spectrum showed estimates of 7.78% in 2018 by end of breastfeeding. Analysis of trends of MTCT by province showed heterogeneity across and within provinces (data not shown) [27]. Layering of survey and MOHCC data shows that similar geographical areas are hot spots for MTCT (Figure 3). MOHCC data includes data from all ten provinces, showing regions where it is most critical to intervene.

Risk factor analysis in the 2018 survey (which covered five of ten provinces) found that a higher prevalence of partner accompaniment for first ANC was associated with a decrease in MTCT, as was knowledge of an HIV‐positive status before pregnancy and receipt of antiretroviral prophylaxis for the baby, Table 2. Women who travelled (received care at more than one facility) more than doubled the risk of MTCT. Of note, out of 25 MTCTs in the 2018 survey, 12 were among MB pairs where the mother had previously tested HIV negative. Programme data on HIV Positive Child Case Investigation found that the majority of mothers that transmitted HIV to their infants booked for ANC late, with a median of 23.5 months, and 40% were unaware of their partner’s HIV status, Table 3. Additionally, 53% of infected babies were born to mothers who were reported to be negative before pregnancy. Case investigation data also demonstrate MTCT was explained by late HIV diagnosis and limited time on ART among mothers before delivery. In addition, as reported in all survey rounds, the case investigation process revealed that maternal mobility increased transmission risk.

**Table 2:** Univariable analysis of factors associated with MTCT in 2018 survey

Factor	Number (%) MTCT	Odds ratio (95% confidence interval)	*p*
Timing of ANC registration/month	–	1.20 (0.96‐1.52)	0.11
Partner accompaniment to ANC			
No	14 (5.45)	1	0.01
Yes	3 (1.30)	0.23 (0.065‐0.81)	
No partner	1 (25.00)	5.79 (0.56‐59.25)	
HIV status before pregnancy			
Negative	14 (6.6)	1	0.01
Positive	7 (2.1)	0.32 (0.13‐0.81)	
Baby received ARV prophylaxis			
No	14 (7.9)	1	0.003
Yes	11 (2.4)	0.29 (0.13‐0.65)	
Received care at more than one facility			
No (one facility)	11 (2.6)	1	0.03
Yes	12 (6.5)	2.55 (1.10‐5.89)	

ANC, antenatal care; ARV, antiretroviral; MTCT, mother‐to‐child transmission.

**Table 3:** Descriptive analysis among case investigation form respondents (N = 271 HIV‐positive infants)

Factor	Number (%) or parameter
Timing of ANC registration/month	Median 23.5 weeks/5.4 months (N = 96)
Male partner HIV status	
Negative	20 (7.4)
Positive	104 (38.4)
Unknown	109 (40.2)
Not Documented	37 (13.7)
HIV status before pregnancy	
Negative	143 (52.7)
Positive	81 (29.9)
Not documented	47 (17.3)
Baby received ARV prophylaxis	
No	67 (24.7)
Yes	166 (61.2)
Not documented	38 (14.0)
Received care at more than one facility	48/219 (21.9) ‐ maternal mobility noted in free text comments

ANC, antenatal care; ARV, antiretroviral.

**Figure 4:**
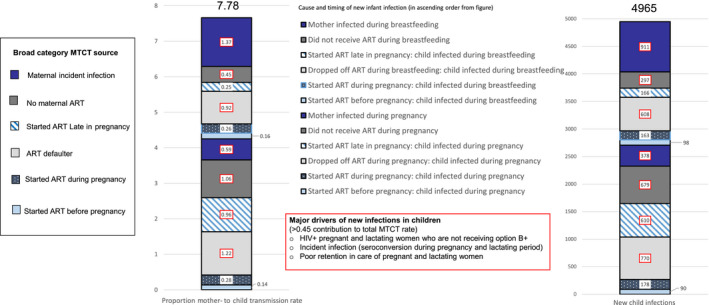
Modelling of MTCT rate by source. ART, antiretroviral therapy; MTCT, mother to child transmission.

Of note, modelling indicated that the majority of MTCTs are attributable to mothers who become infected during breastfeeding (Figure 4), pointing to the need to strengthen HIV‐retesting, risk screening, primary prevention and follow‐up care of HIV‐negative mothers postnatally (in addition to the care for HIV‐positive mothers which has been more optimally given).

A key finding is the variable coverage and completeness of MTCT case investigation: some health facilities complete this comprehensively for all newly diagnosed infants, while in many facilities there were gaps which did not allow elucidation of the cause of transmission.

**Figure 5:**
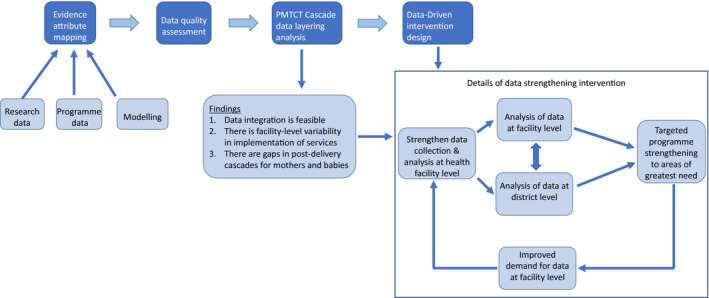
Process of data integration and summary of findings and resulting intervention.PMTCT, prevention of mother‐to‐child transmission.

The data integration process led to decisions on how programmes/data systems could be improved. See Figure 5 and Boxes 1 and 2 for examples of such decisions/effects.

Box 1 **Data integration leads to programme strengthening – maternal mobility during perinatal care and risk of postnatal transmission**

Preliminary analysis of the *2018 survey* data showed that MTCT was higher in the group who visited >1 facility (6.5% vs. 2.6%, odds ratio 2.55 (95% confidence interval 1.10‐5.89), *p* = 0.03).

*MOHCC HIV Positive Child Case Investigation Forms* documenting maternal and infant characteristics of new paediatric diagnoses were submitted by 118/669 OPHID‐supported sites from January 2018 to September 2019 and were electronically entered and analysed centrally. During form review, free text comments reported maternal travel during antenatal/postnatal HIV care among 21.9% (48/219) of HIV‐positive women who transmitted to their infants 0‐24 months.

Travel during infant exposure period has been recommended as a standardized indicator on a revised *MOHCC HIV Positive Child Case Investigation Form* for implementation at all health facilities in Zimbabwe.

Based on these findings, OPHID is working with MOHCC to implement the Strengthening of Information Systems for Elimination of MTCT (SISTEM) – to strengthen PMTCT Programme implementation fidelity and documentation in high MTCT incidence health facilities. SISTEM includes routinely asking and documenting travel plans during the antenatal and postnatal period and strengthened referral systems for women reporting an intention to travel. OPHID is also contributing to the development of a standardized MOHCC Differentiated ART Service Delivery model for mobile and migrant populations, with special considerations for pregnant and lactating mothers.

Box 2 **Data integration leads to programme strengthening – postnatal MTCT is increasingly important**

*UNAIDS SPECTRUM modelling* in Zimbabwe (and globally) suggests that 40% of transmissions are occurring postnatally during breast feeding with a substantial proportion of infections among mothers who were HIV negative at the time of delivery (Figure 4). However, to date there has been limited empirical evidence to support this. In the *2018 PMTCT impact evaluation survey* 12 of the 25 transmissions (48% 95% CI 28.4‐67.6) identified had occurred in mothers reporting that they were HIV negative in ANC.

Furthermore, analysis of *MOHCC HIV Positive Child Case Investigation Forms* in OPHID‐supported facilities revealed that 23% (61/271) of mothers that transmitted HIV to their infants were only diagnosed in the postnatal period.

MOHCC has introduced a *post natal MB Pair register with electronic data entry into DHIS2* which tracks all mother infant pairs from 0 to 24 months postnatally to ensure timely retesting, retention in HIV prevention and care and final outcome ascertainment of both HIV‐positive and HIV‐negative MB pairs. Entry and analysis of MB service uptake and outcomes will be critical for informing PMTCT programme efforts as Zimbabwe approaches EMTCT.

## 4 DISCUSSION

We describe a process for integrating data from different sources to evaluate the PMTCT programme and formulate interventions for strengthening both the data and implementation processes. We found that across datasets, ANC coverage is high, although women generally present late. Uptake of HIV testing among women who present to health facilities is near universal. There are gaps in viral load monitoring of mothers, which may impact MTCT rates. There is variability in PMTCT programme success, with clear MTCT hot spots identified. Investigation of MTCT cases is a recently introduced intervention; we found that this intervention has not yet been adopted across all sites and those sites that implement do so with variable fidelity. Risk factor analysis of MTCT found that late ANC registration with corresponding delay in initiation of ART was critical. Other factors associated with MTCT include mobility of mothers and accompaniment to HIV testing or ANC by partners. Of note, estimates of MTCT differ between Spectrum and survey data (7.78% and 3.8% in 2018), likely because the survey measured MTCT up to a median of 11.5 months postnatally whereas Spectrum estimates MTCT at cessation of breastfeeding. Both modelling and research data suggest that the largest source of MTCT is among women who have tested HIV negative during ANC but who seroconvert and transmit during breastfeeding, but there are currently no programme data showing follow‐up of HIV‐negative women postnatally.

**Figure 6:**
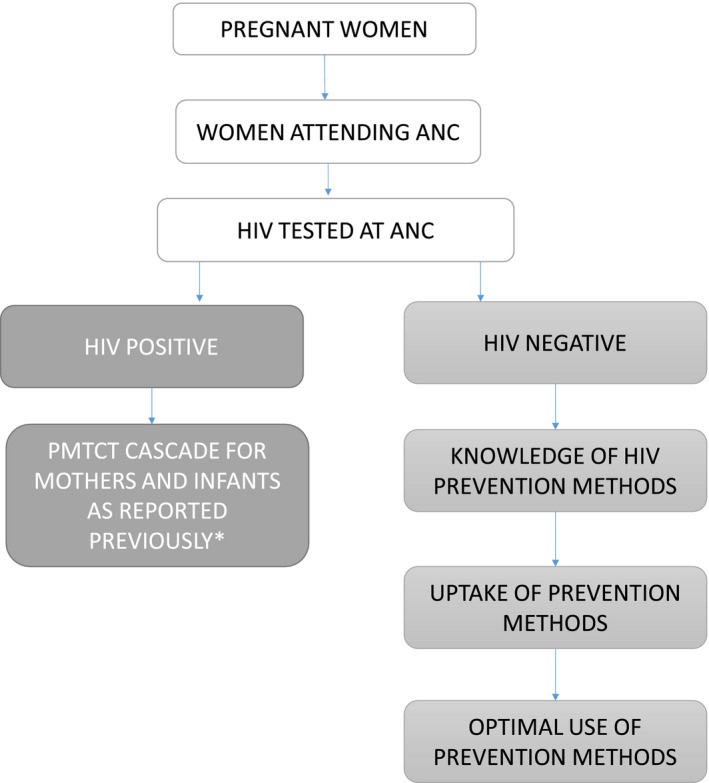
Expanded PMTCT Cascade to include the prevention cascade among HIV‐Negative Women.*Hamilton *et al*., JAIDS 2017. ANC, antenatal care; PMTCT, prevention of mother‐to‐child transmission.

Lack of follow‐up data of HIV‐negative women in the face of high HIV incidence in this group calls for strengthening of implementation and documentation of prevention interventions in this group: there is need to increase the demand, supply and optimal use of both retesting and prevention methods among HIV‐negative women postnatally. Women need to receive information/education on existing prevention methods, with tailored messaging according to type of woman, for example, young women may be told about mentored mothers programmes [28] while other women may benefit from PrEP or circumcision of their partners. The PMTCT cascade would therefore need to be extended to capture engagement with prevention: (i) how many women know of prevention methods; (ii) how many took up prevention methods, and, (iii) how many optimally used the methods; see Figure 6. In pursuit of ensuring better follow‐up of HIV‐negative women MOHCC are currently rolling out guidelines to routinely support and document engagement of individual MB pairs to 24 months postpartum regardless of maternal HIV status. All MB pairs living in a facility catchment area are entered into a mother infant care register to facilitate tracking and early identification of loss to follow‐up, with data entered electronically into DHIS2. This process could be strengthened through training and mentoring of health workers to implement with fidelity and regularly review and act on their programme data to optimize maternal retention in the primary prevention cascade of recommended services.

Similarly, facilities need to be supported to improve and act on results of their MTCT case investigation to provide learning on where gaps/bottlenecks are. Although MOHCC has introduced MTCT case investigation it has not yet been widely implemented. Health facilities need capacity building to strengthen their use of data to ensure timely improvements in implementation, which may include training and mentorship as well as providing feedback on performance. In addition, given the negative impact of mobility of women during pregnancy on MTCT, interventions to strengthen engagement and ensure between facility referral are being considered. Together with MOHCC, OPHID are planning to pilot a differentiated service delivery model for mobile and migrant pregnant and lactating women living with HIV, which includes data strengthening for documentation of referrals and confirmed uptake.

There is need to promote early registration for ANC. Previous qualitative research in Zimbabwe has shown that although demand for ANC among women is high, they may face personal/family barriers such as fear of HIV testing and lack of male partner support [29], and supply‐side barriers such as reluctance to engage with unfriendly health workers. Many suggestions on how uptake of ANC can be improved have been made [29], including improvement of male partner support and removal/abolition of user fees [30], which MOHCC has adopted.

The strengths of this paper include the comprehensive data that comes from four sources, giving us deeper understanding of the PMTCT programme in Zimbabwe. Combining data from different sources potentially allows us to overcome the inherent limitations/weaknesses of each individual data source. For example, while survey data on timing of testing and engagement of services are limited by self‐reporting, programme data are generally objectively (if incompletely) collected. Our survey data have robust numerators and denominators, while programme data have incomplete data on denominators. The systematic process by which we conducted the integration/triangulation exercise gives us confidence in the results. Also, the triangulation process has potential utility for extrapolating missing data, which may prove important when data are not immediately available.

Limitations of the data integration exercise include the use of different sampling and data collection methods, with sampling occurring at different time periods which limits the ability to compare with certainty. The quality and completeness of data varied by data source. Indicators were not always measured in the same way (for example some were measured through self‐report during the survey but by clinic record from programm). Spectrum estimates of post‐partum transmission relied on transmission rates pre‐ART. Although there was overlap of geographic regions covered in many instances, in some cases there was poor or no overlap. Despite all these weaknesses, integration ensured that weaknesses in one data source were compensated for to a certain extent by the other sources, and we showed similar findings where data across sources were available.

## 5 CONCLUSIONS

By systematically integrating data from multiple sources, a number of areas for PMTCT programme strengthening were identified. In addition, important data gaps became apparent. The data integration working group is developing a package of data strengthening interventions informed by this work for rollout and evaluation and proposes that the cascades be extended to fully capture PMTCT and maternal and infant survival.

## COMPETING INTERESTS

No competing interests are declared.

## AUTHORS’ CONTRIBUTIONS

ELS, KW, FMC, SM, NP and AM formulated the research study and design. CW, JD, MD, IT, AC, KW, AM and SM collected data and informed design of data collection methods. IT, AC, MD, JD, MKD, CF and SIM analysed data or contributed to analysis. ELS and KW wrote the first draft of the manuscript. FMC, KW, ELS, NP, EG, SIM, CF, MKD, SM and AC substantial intellectual input to manuscript.

## Appendix 1

**Table A1:** Stage 2: template for assessing data quality – adapted from Kahn *et al*. [26]

Data Source	Category	Sub category	Verification checks	Validation checks	Comments and overall assessment of quality
Data source (complete for each data source)	**Conformance** Do data values adhere to specified standards and formats?	Value conformance (i) whether data values conform to internal formatting constraints or (ii) whether they conform to allowable values or ranges Relational conformance (i) whether data values conform to relational constraints; (ii) key unique data are not duplicated Computational conformance – computed values conform to computational/programming specifications			
	**Completeness** Are data values present?				
	**Plausibility** Are data values believable?	Uniqueness plausibility – data values that identify a single object are not duplicated Atemporal plausibility – agreement with internal measurement or local knowledge, independent measures of the same fact are in agreement; repeated measures of the same fact show the expected variability Temporal plausibility – conformance to expected temporal properties			
	**Relevance** Are the data relevant to current programme practice – scale, recency				
